# 1Click1View: Interactive Visualization Methodology for RNAi Cell-Based Microscopic Screening

**DOI:** 10.1155/2013/156932

**Published:** 2012-12-27

**Authors:** Lukasz Zwolinski, Marta Kozak, Karol Kozak

**Affiliations:** ^1^Nauru, LTD., Breslau, Poland; ^2^Technical University of Dresden, 01307 Dresden, Germany

## Abstract

Technological advancements are constantly increasing the size and complexity of data resulting from large-scale RNA interference screens. This fact has led biologists to ask complex questions, which the existing, fully automated analyses are often not adequate to answer. We present a concept of 1Click1View (1C1V) as a methodology for interactive analytic software tools. 1C1V can be applied for two-dimensional visualization of image-based screening data sets from High Content Screening (HCS). Through an easy-to-use interface, one-click, one-view concept, and workflow based architecture, visualization method facilitates the linking of image data with numeric data. Such method utilizes state-of-the-art interactive visualization tools optimized for fast visualization of large scale image data sets. We demonstrate our method on an HCS dataset consisting of multiple cell features from two screening assays.

## 1. Introduction

High Content Screening (HCS) is of growing importance, allowing for efficient large scale experiments in biological research including drug discovery [1, 2]. HCS uses microscopy images of cells and advanced image analysis methods to detect the effects of RNAis or small chemical compounds on a cellular process of interest; in a multivariate approach, multiple cell parameters are collected, allowing for a complex analysis approach. HCS data include information about the library of bioactive molecules, up to several million microscopy images as well as image analysis data with more than 100,000 rows and more than 20 columns (matrix: RNAi sample × cell features). For quantified image processing results, there is a need to generate statistical, quality control and bioinformatics information up to the final hit list. The use of any data analysis tool requires the researcher to appropriately tune these parameters for a specific dataset, in order to avoid arbitrary results in terms of the number of data clusters, size, or density.

Today, HCS experiments are routinely performed under multiple experimental conditions on multiple test samples and with multiple staining channels. For example, in an experiment with two genotypes and two time points, a scientist might be interested in finding genes that are similarly expressed at the first time point in both genotypes but expressed differently at another point in the genotypes. Exploring such data can benefit substantially from interactive visualization tools that bring the problem of data mining and analysis closer to the individual researcher in the field, by allowing real-time visual data manipulation.

Applied to the context of this paper, HCS data analyses belong to information visualization. Shneiderman [3] published the often-cited Visual Information Seeking Mantra: “Overview first, zoom and filter, then details on demand.” Data visualization played an important role already in early reports in cancer microarray studies. For instance, Khan et al. [4] summarized their analysis results in a planar visualization that shows a clear separation of diagnostic cases. VizRank [5] can score the visualizations according to the degree of class separation and can work through projection candidates to find those with the highest scores. Differently to the approach proposed in this paper, information in their visualization could not be traced back to the original genes as the plot was obtained by multidimensional scaling and used features crafted in several data preprocessing steps (feature selection through neural network learning and feature construction by principal component analysis). Their visualization was therefore not a result of an explicit search. McCarthy et al. [6] were the first to show how RadViz algorithm which can be applied to the analysis of class-labeled datasets from biomedicine. They focused on feature subset selection to reduce the number of genes in visualization and feature grouping (clustering anchors of correlated features) and showed that in such an arrangement the visualizations can provide for a clear separation of instances of different class. We present here unique software methodology which not only provides interactive visualization of data but also links quantified image processing results with raw microscopic image data based on KNIME workflow environment [7].

Sharing requires centralized-image databases repositories which require common image data formats. This is particularly challenging for multidimensional microscopy image data, which are acquired from a variety of microscopes. 1Click1View (1C1V) method uses the sophisticated open standard format Bio-Formats that supports different types of screening image data (e.g., Tiff 8bit, 16bit, JPEG, JPEG2000, etc.). A user can easily access basic image processing functions from image viewer.

In order to facilitate data preparation for visual analysis, preprocessing and postprocessing steps should be simple and should not require programming knowledge. We suggest to implement 1C1V in workflow systems which is crucial for enabling users to deal with data preprocessing and post-processing. The concept of workflow is not new, and it has been used by many organizations, over the years, to improve productivity and increase efficiency on data preparation. A workflow system is highly flexible and can accommodate any changes or updates whenever new or modified data and corresponding analytical tools become available. 1C1V is a general-purpose interactive visualization methodology for multiparametric screening assays that is easy to integrate with KNIME [7] workflow system. The basic idea of 1C1V is to provide the data in one visual frame that allows users to gain insight into the data and generate hypotheses by directly interacting with image data. The advantage of 1C1V is that users are directly involved in the image processing results to combine the flexibility, creativity, and general knowledge of the scientist with the enormous amount of numerical rows connected to image files. 1C1V is developed to help scientists and answer complex queries through interactive visual exploration of screening datasets. One key attribute of the 1C1V that distinguishes it from past and current image data exploration methodologies is that the original image data, their image processing results, and metadata (additional information captured by acquisition software about an image, such as the instruments used, camera, acquisition settings, image size, and resolution) are linked together and are available for filtering, clustering in interactive view. 

## 2. Systematic Errors and Quality Control in HCS

HCS has already proven successful as a method to deliver more relevant information simultaneously in one experiment, rather than delivering a single readout in a series of sequential experiments [8–11]. A prototype scenario might be the series of simultaneously available readouts obtained from a cellular assay. One parameter identifies cells (i.e., membrane dye at first wavelength), another determines the stage of mitotic change (e.g., fragmented and condensed nuclei at a second wavelength), and a third parameter classifies the apoptotic stage using morphological criteria at a third wavelength. Certainly, these analyses can already be performed almost autonomously with very high throughput. HCS operates with samples in microliter volumes that are arranged in two-dimensional plates. A typical HCS plate contains 96 (12 × 8) or 384 (24 × 16) samples. The quality control and normalization procedure in plate-based primary HCS screens can be mainly performed by using interactive visual analysis. 

Quality of measurements has a number of advantages, including objectivity, reproducibility, and ease of comparison across screens. Random and systematic errors can cause a misinterpretation of candidates to be a hit. They can induce either underestimation (false negatives) or overestimation (false positives) of measured parameters. Various methods dealing with quality control are available in the scientific literature. These methods are discussed in details in the papers by Brideau et al. [12], Heyse [13], and Zhang et al. [14, 15]. There are various sources of systematic errors:

- Systematic errors caused by ageing, reagents evaporation or decay of cells can be recognized as smooth trends in the plate's means/medians;
- Errors in liquid handling and malfunction of pipettes can also generate localized deviations of expected data values;
- Variation in incubation time, time drift in measuring different wells or different plates, and reader effects may be recognized as smooth attenuations of measurement over an assay.

Brideau et al. [12] demonstrated examples of systematic signal variations that are present in all plates of an assay. For instance, Brideau et al. [12] illustrates a systematic error caused by the positional effect of detector. To guarantee this reliability avoiding systematic errors, data quality control at different levels is a must. This begins in the optimization phase of the assay: In test runs with a small number of compound plates, the assay has to possess a sufficient signal window (e.g., *Z*-factor [15]), stability, and sensitivity (e.g., measured by the effects of known control compounds) [16, 17]. If problems occur, the parameters of the assay or even its format should be tuned to match the quality criteria of HCS. Data quality control on the level of an individual assay seeks again to guarantee assay stability and sensitivity, which must be monitored constantly using the appropriate controls. At the same time, it tries to pick up on process artifacts caused by failures in the screening machinery or the test system (e.g., a blocked pipettor needle, air bubbles in the system, or a changing metabolic state of reporter cells) [13].

If unnoticed, these artifacts can result in a high number of false positives, but seemingly “highly specific hits”. Often, such process artifacts can be detected by changes in the overall signal or by specific “signal patterns” on plates (e.g., pipettor line patterns), if the compound library is randomized across the screening plates. Visual analysis of QC with link to images is preferably done directly after the screening run to ensure that such patterns can be traced back to their origin (e.g., the pipettor may be inspected the next morning) and can be unambiguously classified as artifacts—or nonartifacts. This distinguishes false positives from real actives that should be more or less randomly dispersed when considering a whole series of plates from reasonably randomized compound collections. 

**Figure 1:**
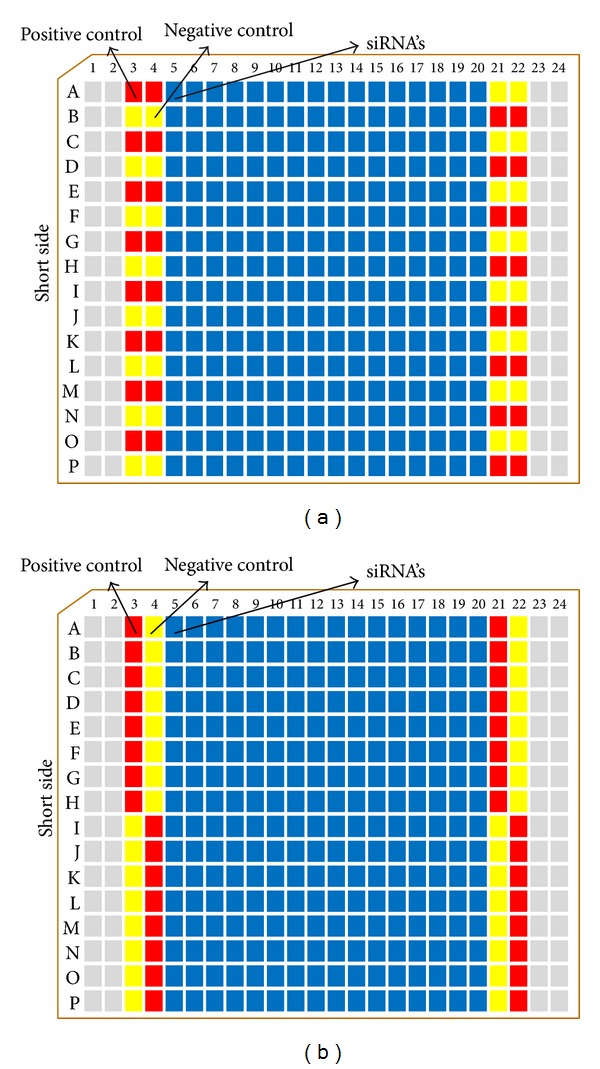
Location of controls on a 384-well plate. In a screening process, the designed biological assay is performed by using a robot to add the cell line and specific reagents (siRNA) to each well, which already contains a different oligonucleotide or control. After incubation or other required manipulations, fluorescence images are acquired and obtained for every well by an automated microscope. These raw data represent the images of each oligonucleotide or control against a specified target. (a) Generally, in a siRNA, 256 different oligonucleotides (blue) are stored in the middle of a 384-well plate, and wells on the first two and last two columns are left empty. (b) Ideally, controls should be located randomly among the 384 wells of each plate. Only the first two and the last two columns are typically available for controls. Despite this limitation, edge-related bias can be minimized by alternating the sixteen positive controls (red) and the sixteen negative controls (yellow) in the available wells, such that they appear equally on each of the sixteen rows and each of the 4 available columns.

The hypothesis underlying HCS data analysis is that the measured image descriptors for each single siRNA represent its relative number of observed objects at fluorescence image. Within-plate reference controls are typically used for these purposes (Figure 1). Controls help to identify plate-to-plate variability and establish assay background levels. 

## 3. Interactive Image Data Exploration in Workflow Environment

**Figure 2:**
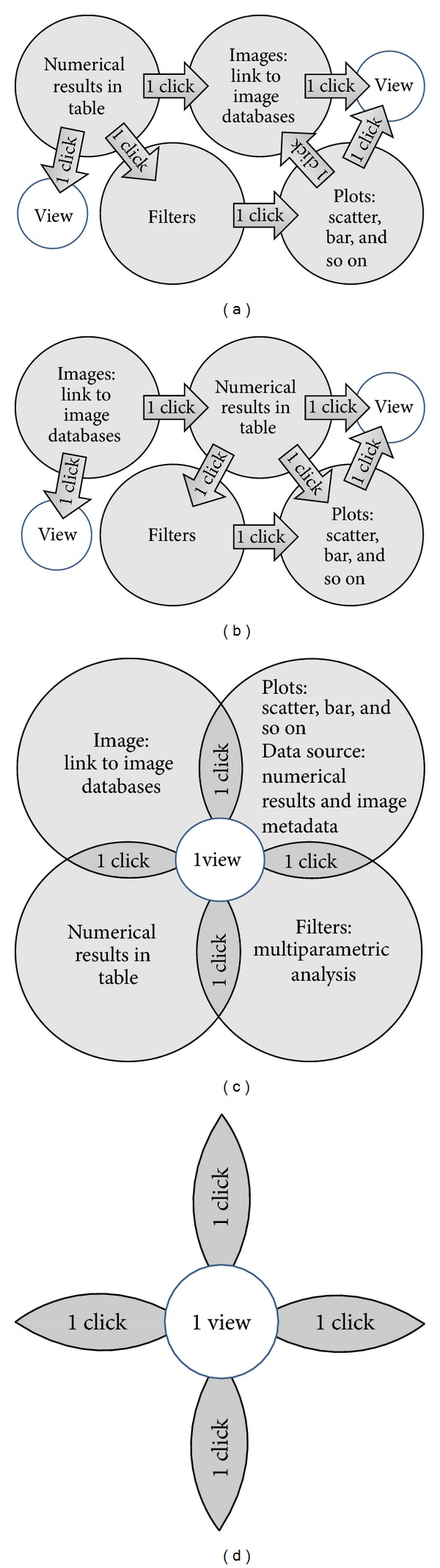
(a) and (b) traditional analytic scenarios for image-based datasets, multiple clicks, and distributed views. (c) 1C1V method: using one view and with one click scientist should get access to all related data sources and analytical tools: images, plots, numerical results, and filters. The concept is the focus to reduce number of clicks—access to all information should be direct, and user actions should be minimized with maximum of interactions. (d) Star model as software methodology for interactive visualization tools.

High dimensionality of datasets makes it difficult to find interesting patterns. To cope with high dimensionality, low dimensional projections of the original dataset are generated; human perceptual skills are more effective in 2D or 3D displays. Mechanisms have been developed to enable researchers to select low dimensional projections, but most are static or do not allow users to make one view frame and crosslink what is interesting to them. Software applications based on those mechanisms require from users many independent clicks which in consequence change the content of main view panel (Figures 2(a) and 2(b)). There is, therefore, a need for a mechanism that allows users to choose the property of projections that they are interested in, rapidly examine projections, make filtering, and locate interesting view panels to detect patterns, clusters, or outliers. 

In 1C1V, data can be visualized at several stages of analysis and linked to raw image data keeping concept of star model (Figures 2(c) and 2(d)): one-click, one-view. Unmodified and transformed datasets can be plotted interactively as scatter plots, displayed in histograms, viewed in image viewer/editor or viewed as tables. Entire experiments can be displayed in various overview plots in the context of how they are annotated, and figures and tables can be exported for publication. Data can also be exported for custom analyses (e.g., for algorithms that are very expensive to computer power and time) and local development of new analysis methods and in various defined formats for use in external postanalysis applications. 

**Figure 3:**
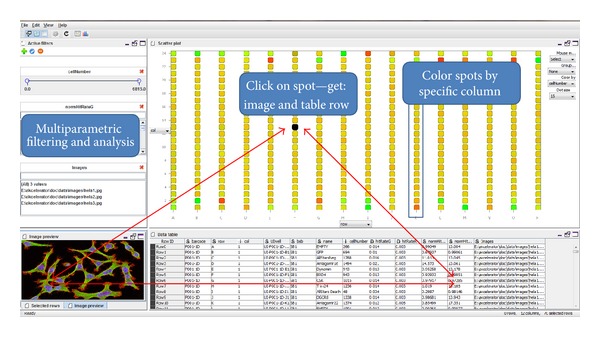
Main parts of the 1C1V as prototype implementation of interactive user interface. One click on spot or table row, or image has visual consequences on all GUI panels of one software view.

Data can be visualized at several stages of analysis. Unmodified images and extracted results datasets can be plotted interactively as scatter plots, displayed in histograms, or viewed as tables (Figure 3). An entire result table can be displayed in various overview plots, and tables can be exported for publication. From any data-analysis step, the experiment can be imported into a data-visualization interface in in which the data can be browsed and viewed.

Tools based on 1C1V method should provide a number of projections which interact with image data, filters, tables, and colors (Figure 3). First step to be done after loading the data (images) and extracting image features is configuring GUI, that is, deciding which variable is associated with which axis. This configuration is done in real-time and can be changed easily. Once the axes are set and the data is mapped to the unit cube, the user can start to explore and identify interesting objects by performing the following operations.

1. Create 2D scatter plots of any two experiments by orienting the third axis perpendicularly to the display plane.
2. Select “interesting” objects (e.g., genes) and filter out uninteresting objects (e.g., genes) using a combination of selection tools.
3. Assign color and shape to selected objector groups of genes.

Nauru offers four different ways to select objects. They are as follows.

1. Mouse Click: a gene can be selected by a mouse click. Annotation for the selected gene and its parameter values for all experiments are shown.
2. Selection Plane: the selection plane is a 2D rectangle. A user can move and resize the rectangle. All genes that lie within the rectangle after projection on to the 2D screen are selected. A user can browse through the list of annotation and expression value information of selected genes.
3. Range Selection: a range of differential cell parameters values can be specified for each of the axes and color. All genes falling outside the specified range are not shown. This selection mechanism is useful for setting cut-off values at which the differential expression can be considered significant.

Visualizations are the key to analyzing data in 1C1V. A variety of visualization types can be used to provide the best view of the data:

### 3.1. Image Viewer (IV)

**Figure 4:**
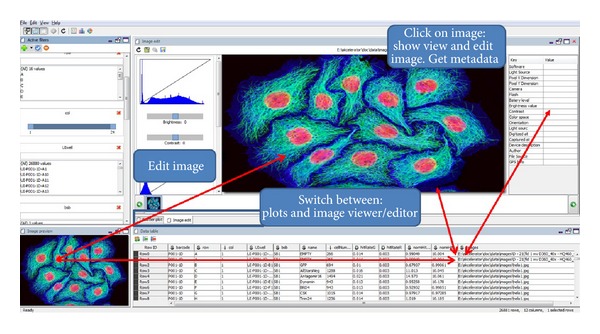
View and edit images in interactive mode. One click on image or table row, or image has visual consequences on all GUI panels of one software View.

The 1C1V image viewer (Figure 4) as a key element for HCS data allows users to visualize and edit images and image processing results. IV is highly integrated with interactive table (e.g., sample annotation), plots, data visualization features, and data controls. Image Viewer displays images very quickly, and these images may be viewed in full screen, as slideshows or as thumbnails. It is quite capable of processing images; user can rotate, adjust brightness and color, and apply filters or LUT tables for each independent staining channel. The editor of image viewer has a variety of tools like a Fire, 3-3-2 RGB, Grays, Ice, Spectrum, and export figures for publication and great effects that can be applied on selected image or entire image collection. Moreover, thanks to Bio-Formats plugin, it reads many formats, including Tiff: (8, 16, and 32 bits), JPEG, and JPEG2000. A very special feature of image viewer is a raw data security system. All image processing steps are saved as independent feature mask in settings file. Raw images stay unchanged. Batch processing options (apply the same image processing steps for all images) can be carried out on more than one file at a time by clicking “Save”. It offers nearly instantaneous hotkey zooming. It also allows having several running multiple instances of image panel if you like to browse in different windows.

IV also recognizes image metadata data and optionally displays it alongside the images. Metadata panel can display the basic image metadata like microscope features or exchangeable image file format (exif) tags. Another outstanding plus of IV is the transformation of image metadata into an interactive table (described below). Metadata Panel can also decode CR2, CRW, and exif files and extract the embedded headers. Exif data are embedded within the image file itself. Exif is a standard that specifies the formats for images, ancillary tags used by digital cameras, scanners and other systems handling image, and sound files recorded by digital cameras. The specification uses the following existing file formats with the addition of specific metadata tags: JPEG DCT for compressed image files, TIFF Rev. 6.0 (RGB or YCbCr) for uncompressed image files, and RIFF WAV for audio files (Linear PCM or ITU-T G.711 *μ*-Law PCM for uncompressed audio data, and IMA-ADPCM for compressed audio data). It is not supported in JPEG 2000, PNG, or GIF. The exif format has also standard tags for location information. Currently, few cameras and a growing number of mobile phones have a built-in GPS receiver that stores the location information in the exif header when the picture is taken. 

### 3.2. Scatter Plots

Scatter plots are similar to line graphs in that they use horizontal and vertical axes to plot data points. However, they have a very specific purpose. Scatter plots show how much one variable is affected by another. Each record (or row) in the dataset is represented by a marker whose position depends on its values corresponding to the *X* and *Y* axes.

The above picture demonstrates how scatter plots can be used. Say, for example, that a user wants to show whether there exists a consistency in cell number across all the screening samples. A third variable can be set to correspond to the color or size of the markers, thus adding yet another dimension to the plot. Two-dimensional scatter plots are the default visualization of many datasets.

### 3.3. Bar Charts

A bar chart is a way of summarizing a set of categorical data. It displays the data using a number of bars of the same width, each of which represents a particular category. The length of each bar is proportional to the count, sum, or the average of the values in the category it represents, such as age group or geographical location. In Nauru, it is also possible to color or split each bar into another categorical column in the data, which enables you to see the contribution from different categories to each bar in the bar chart.

### 3.4. Interactive Tables

The table visualization presents the data as a table of rows and columns. The table can handle the same number of rows and columns as any other visualization in Nauru. In the table, a row represents a record. By clicking on a row, you make that record active, and by holding down the mouse button and dragging the pointer over several rows, you can mark them. You can sort the rows in the table according to different columns by clicking on the column headers or filter out unwanted records by using the query devices. Different types of visualizations can be shown simultaneously. They are linked and are updated dynamically when the query devices are manipulated (see below). Visualizations can be made to reflect high-dimensional data by letting values control visual attributes such as size, color, shape, rotation, and text labels. You can sort the vertical order of the rows in the table. This can be done in several steps, for example: first sort, according to the values in column 1, then by the values in column 5, then by the values in column 3, and so forth.

### 3.5. The Filter Panel

Filter panels are used to filter data. Filter panel devices appear by demand in several forms, and scientist can easily select a type of query device that best suits user's needs (e.g., combo boxes, sliders, etc.). When manipulating a filter by moving a slider or selecting a multiple box, all visualizations are immediately updated to reflect the new selection of image data. In such cases, researchers want to quickly find only RNAi constructs, genes, or compounds similar to the expected pattern. 

## 4. Workflow Platform for Preprocessing and Postprocessing

**Figure 5:**
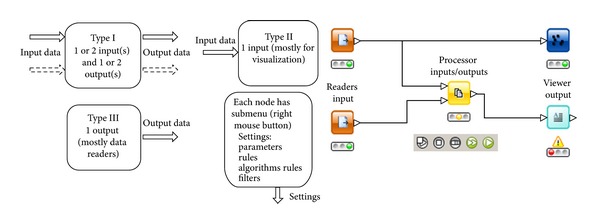
General concept of a workflow framework. The component properties are described by the input metadata, output metadata and user defined parameters or transformation rules. The input and output ports can have one or more incoming, outgoing metadata or images.

The concept of workflow is not new, having been used by many organizations, over several years, to improve productivity and increase efficiency. A workflow system is highly flexible and can accommodate any changes or updates as when new or modified data and corresponding analytical tools become available. Raw metadata are not always in ready format for visualization. A workflow environment allows biologists themselves to prepare data for visualization without involving any programming. Workflow systems are different from programming scripts and macros in one important respect. Programming systems and macros use text-basedlanguages to create lines of code, while applications like Nauru use a graphical programming language. In general, workflow systems concentrate on creation of abstract process workflows to which data can be applied when the design process is complete. In contrast, workflow systems in the life science domain are often based on a dataflow model, due to the data-centric and data-driven nature of many scientific analyses. A comprehensive understanding of biological phenomena can be achieved only through the integration of all available biological information and different data analysis tools and applications. A workflow environment allows HCS researchers themselves to perform the integration without involving any programming. As such, the workflow system allows the construction of complex *in silico *experiments in the form of workflows and data pipelines. Data pipelining is a relatively simple concept. Any computational component or node has data inputs and data outputs. Data pipelining views these nodes as being connected together by “pipes” through which data flow (Figure 5). 

Nauru builds a flow by dragging and dropping nodes from the node repository into the main panel and connecting them. Nodes are the basic processing units of a workflow (Figure 5). Each node has input and/or output ports. Data are transported through connections from these node output ports into connected input ports. After positioning the nodes, the inputs of each node are fully connected to outputs of a predecessor node. This is achieved by clicking on an output port and dragging the connection to the input port that should receive data from this output. All data flowing between nodes are wrapped within a class called DataTable, which holds metainformation concerning the column headers and the actual data (e.g., numeric data, image name, image path, image processing parameters, and gene annotations). The data can be accessed by iterating over instances of DataRow. Each row contains a unique identifier (or primary key), a specific number of DataCell objects, image name, and image path. A workflow system is highly flexible and is designed to accommodate any changes on table before interactive visualization.

## 5. Comparison of Existing Visualization Methods in HCS

We compared existing solutions for visualizing of HCS data and made a summary and comparison of few methodologies and solutions in Table 1.

## 6. Case Studies

Visualization of image processing features extracted from raw data helps to discover systematic plate-to-plate variation, making measurements comparable across plates. Systematic errors decrease the validity of results by either over- or underestimating true values. These biases can affect all measurements equally or can depend on factors such as well as location, liquid dispensing, and signal intensity. Although recent improvements in automation can minimize bias, and thereby provide more reproducible results, equipment malfunctions can nonetheless introduce systematic errors, which must be corrected at the data processing and analysis stages. For illustration, two screens have been taken into visual quality control analysis using Nauru software described in Table 2. For each cell of the samples, the image processing software (Advanced Cell Classifier [18]) quantified the images by calculating more than a hundred parameters, which mainly fall into the following categories:

- geometric properties such as the area, perimeter, and shape of the cell nucleus; the location of a cell; average distance of a cell to its neighbours;
- intensity information such as the content of a protein, as reflected by the intensity of the corresponding fluorescent dye, and the variance, skewness, and kurtosis of the intensity distribution.

**Figure 6:**
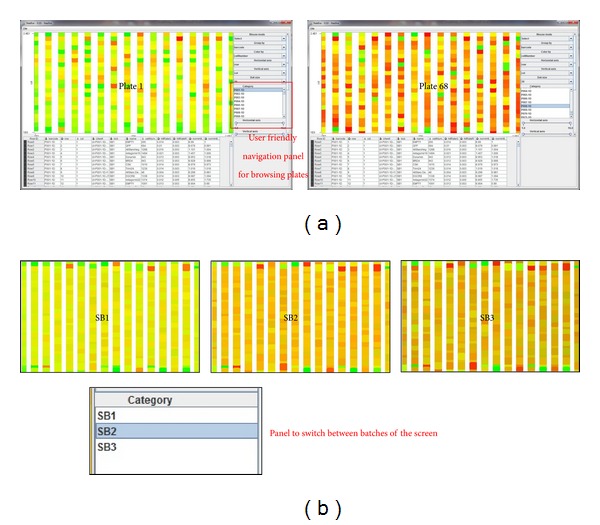
(a) Usage of right panel in order to detect a problematic plate 68 in screen 1. (b) Observation to see consistency between batches of the screen 2 (screen was done in 3 × 25 plates batches). In same view it is easy to detect microscopic lamp intensity reduction over the full screening run (important for normalization method). We can also observe on batch SB1, SB2, SB3 very strong edge effect.

**Figure 7:**
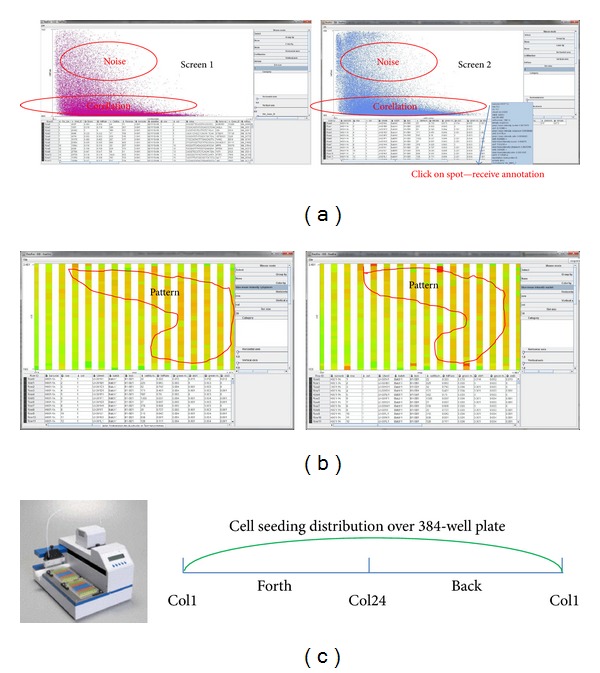
(a) Screens 1 and 2: hit rate to cell number correlation. (b) Detection of cell culture seeding pattern produced by cell seeder in screen 2. (c) Cell seeder and seeding distribution over 384-well plate.

As a result of the analysis of screens 1 and 2, we were able to detect significant errors:

- plate-to-plate variation (Figure 6(a)),
- problematic plates (Figure 6(a)),
- edge effect on row A and P of 384 well plate (Figures 6(a) and 6(b)),
- signal intensity reduction over plates (Figure 6(b)),
- batch/sub-batch consistency (Figure 6(b)),
- hit-cell analogy assessment (Figure 7(a)),
- mistake on dispensing-cell seeding (Figure 7(b)),
- cell seeder and seeding distribution over 384 well plates (Figure 7(c)).

## 7. Conclusion

In this paper, we offer interactive methodology 1C1V for the analyses of HCS datasets. High dimensionality of the datasets hinders users from recognizing important patterns in the datasets. We add a scatter plot browsing mechanism that helps users select interesting 2D projections of the high dimensional multiparametric dataset. In addition, an even more difficult problem is to understand the biological significance of the patterns found in the datasets. Considering the needs of working professionally in the field of HCS data analysis, 1C1V is an effective and efficient method for interactive image data exploration, detection of systematic errors, and quality control. We have demonstrated through simple examples how quickly a researcher could investigate problems of a dataset before making a final decision. 

This work is a part of our continuing effort to give users more controls over data mining processes and to enable more interactions with analysis results through interactive information visualization techniques. These efforts are designed to help users perform exploratory data analysis, establish meaningful hypotheses, and verify results. In this paper, we show how those visualization methods can help molecular biologists analyze and understand multidimensional RNAi cell-based screening data. Currently for licensing reasons a tool Nauru developed based on 1C1V methodology is not available as open source software. At the beginning of year 2013, the open source version of 1C1V will be available as KNIME node [7].

## Figures and Tables

**Table 1:** Selected visualization methodologies in HCS.

Method and solution	Description	Reference
Visual data exploration (VDE)	The basic idea of VDE is to present the data in some visual forms that allow users to gain insight into the data and generate hypotheses by directly interacting with the data. The advantage of VDE is that users are directly involved in the data mining process to combine the flexibility, creativity, and general knowledge of the human with the enormous storage capacity and the computational power of computers. This process is especially useful when little is known about the data and the exploration goals are vague, such as in analyzing a huge number of RNAi-HCS images. However, access to plots, data tables, and image viewer in one view frame available with one click are not available.	[19]

Cellomics Discovery ToolBox and visualization method	The methods focus on visualizing simple quantitative readouts of markers instead of images and especially the relationships among images that convey profound information closely related to effects of chemical compounds, gene functions, and biological processes.	http://www.cellomics.com and [20]

PhotoFinder and Personal Photo Libraries	Those methods focus on image database visualization targeted at personal photo albums, which are much smaller than HCS image databases and did not consider computational needs specific to HCS image analyses.	[21, 22]

ImCellPhen—interactive mining of cellular phenotypes	This is a method and a tool for interactive mining of cellular phenotypes which provides intelligent interfaces for visualizing large-scale RNAi-HCS image databases and interactive mining of cellular phenotypes. However, this method does not provide easy-to-use (with one click access) filtering functionality for image properties and image processing results.	[19] http://combio.cs.brandeis.edu/imcellphen/

The Open Microscopy Environment (OME)	OME provides an open-source browser to navigate HCS image databases that are described as a quasi-hierarchical structure representing the relationship between projects and datasets. However, this navigation scheme was not designed to facilitate discovery of screening hits among all available parameters and categories of data.	[23]

Advanced Cell Classifier	Advanced Cell Classifier (ACC) is a data analyzer method program to evaluate cell-based high-content screens. The basic aim is to provide a very accurate analysis with minimal user interaction using advanced machine learning methods and visual learning of image data sets. However, ACC do not provide full interactivity, and at same time, filtering options for 3 data sources on same view: library, images, and image processing results.	[24]

**Table 2:** Key features of the datasets used in the experiments.

Screen	Description
(1) Biogenesis Screen (BGS)	This screen studied the biogenesis of the small ribosomal subunit in human cells (40S subunit). Towards this, an assay for visual detection of nuclear maturation defects of the 40S subunit was developed. In the cellular process, a HeLa cell line bearing a fluorescently tagged ribosomal protein of the 40S subunit (Rps2-YFP), which is expressed only upon induction, was used to selectively visualize newly synthesized 40S subunits. This assay has been developed to detect nuclear 40S maturation defects upon depletion of a protein by RNAi. This allowed to identify proteins functioning in 40S biogenesis in human cells and, according to the classification, assign their requirement to nucleolar or nucleoplasmic maturation events. Screening results data for QC: 76747 data points corresponding to individual siRNA oligonucleotide (4 siRNAs per targeted gene), including 4 numeric parameters and gene/siRNA annotation.

(2) miRNA biogenesis-Antagomirs Screen	The goal of the screen was to monitor the levels of two individual miRNAs (mir16 with GFP and mir22 with mCherry) within the cells and to investigate in modulators of the miRNA expression/function. In addition, the experiment was designed to study an inhibitor of miRNAs (Antagomir)—interesting point was to know how it is taken up and how it does act (which genes are important). Screening results data for QC: 26880 data points corresponding to pooled siRNAs (4 oligonucleotides per targeted gene), including 5 numeric parameters and gene/siRNA annotation.
